# Primate cerebellar granule cells exhibit a tonic GABA_A_R conductance that is not affected by alcohol: a possible cellular substrate of the low level of response phenotype

**DOI:** 10.3389/fncir.2013.00189

**Published:** 2013-11-26

**Authors:** Claudia Mohr, Olena Kolotushkina, Joshua S. Kaplan, John Welsh, James B. Daunais, Kathleen A. Grant, David J. Rossi

**Affiliations:** ^1^Department of Behavioral Neuroscience, Oregon Health & Science UniversityPortland, OR, USA; ^2^Center for Integrative Brain Research, Seattle Children's Research InstituteSeattle, WA, USA; ^3^Department of Physiology and Pharmacology, Wake Forest School of MedicineWinston Salem, NC, USA; ^4^Division of Neuroscience, Oregon National Primate Research CenterBeaverton, OR, USA

**Keywords:** GABA modulators, GABA-A receptor, alcohol drinking, ethanol, cerebellum, primate

## Abstract

In many rodent brain regions, alcohol increases vesicular release of GABA, resulting in an increase in the frequency of spontaneous inhibitory postsynaptic currents (sIPSCs) and the magnitude of tonic GABA_A_ receptor (GABA_A_R) currents. A neglected issue in translating the rodent literature to humans is the possibility that phylogenetic differences alter the actions of alcohol. To address this issue we made voltage-clamp recordings from granule cells (GCs) in cerebellar slices from the non-human primate (NHP), *Macaca fascicularis*. We found that similar to Sprague Dawley rats (SDRs), NHP GCs exhibit a tonic conductance generated by α6δ subunit containing GABA_A_Rs, as evidenced by its blockade by the broad spectrum GABA_A_R antagonist, GABAzine (10 μM), inhibition by α6 selective antagonist, furosemide (100 μM), and enhancement by THDOC (10–20 nM) and THIP (500 nM). In contrast to SDR GCs, in most NHP GCs (~60%), application of EtOH (25–105 mM) did not increase sIPSC frequency or the tonic GABA_A_R current. In a minority of cells (~40%), EtOH did increase sIPSC frequency and the tonic current. The relative lack of response to EtOH was associated with reduced expression of neuronal nitric oxide synthase (nNOS), which we recently reported mediates EtOH-induced enhancement of vesicular GABA release in rats. The EtOH-induced increase in tonic GABA_A_R current was significantly smaller in NHPs than in SDRs, presumably due to less GABA release, because there were no obvious differences in the density of GABA_A_Rs or GABA transporters between SDR and NHP GCs. Thus, EtOH does not directly modulate α6δ subunit GABA_A_Rs in NHPs. Instead, EtOH enhanced GABAergic transmission is mediated by enhanced GABA release. Further, SDR GC responses to alcohol are only representative of a subpopulation of NHP GCs. This suggests that the impact of EtOH on NHP cerebellar physiology will be reduced compared to SDRs, and will likely have different computational and behavioral consequences.

## Introduction

Alcohol abuse is a leading cause of preventable death and illness, and the economic cost of alcohol abuse, including treatment and healthcare, lost productivity and various adverse social impacts is estimated to be $185 billion annually in the USA alone (Harwood, 2000). Accordingly, a great deal of research is devoted to identifying neural targets of alcohol, and to determining how alcohol actions at those targets impact the brain and contribute to the development of alcohol abuse and dependence (Lovinger and Crabbe, 2005; Enoch, 2008). It has long been thought that a main action of alcohol is to enhance GABAergic transmission, but until recently the specific mechanisms of the supposed enhancement have been elusive (Lovinger and Crabbe, 2005; Enoch, 2008; Helms et al., 2012). Recent studies with Sprague Dawley rat (SDR) brain slice preparations have determined that a dominant mechanism by which alcohol (EtOH) enhances GABAergic transmission is via enhanced vesicular release of GABA, with a resultant increase in the frequency of GABA_A_ receptor (GABA_A_R)-mediated spontaneous inhibitory postsynaptic currents (sIPSCs) (Roberto et al., 2003; Criswell et al., 2008; Theile et al., 2008). The EtOH-induced vesicular GABA release can also increase the magnitude of tonic GABA_A_R currents in cell types that express the specialized class of extrasynaptic GABA_A_Rs that generate tonic GABA_A_R currents (Carta et al., 2004; Hanchar et al., 2005). More controversially, it is also being debated whether the EtOH-induced increase in tonic GABA_A_R current is simply due to the increased vesicular release of GABA and consequent increase in ambient concentrations, or whether there is also a direct potentiation of the GABA_A_R subunits that mediate tonic currents (Hanchar et al., 2005; Borghese et al., 2006; Borghese and Harris, 2007; Botta et al., 2007a,b; Korpi et al., 2007; Santhakumar et al., 2007).

The two forms of EtOH-induced enhancement of GABA_A_R currents are exemplified by cerebellar granule cells (GCs) which express α6δ subunit-containing, extrasynaptic GABA_A_Rs that generate a powerful tonic GABA_A_R-mediated conductance (Brickley et al., 2001; Hamann et al., 2002a; Stell et al., 2003). In electrophysiological studies of SDR cerebellar brain slices, acute application of EtOH increases the frequency of spontaneous GABAergic IPSCs, and increases the magnitude of the tonic GABA_A_-mediated current (Carta et al., 2004; Hanchar et al., 2005; Kaplan et al., 2013). Importantly, in contrast to many brain regions (e.g., hippocampus, nucleus accumbens, thalamus, ventral tegmental area, substantia nigra) (Peris et al., 1992; Nie et al., 2000; Liang et al., 2006; Jia et al., 2008; Theile et al., 2008, 2009), where EtOH only affects GABA_A_R transmission at concentrations far above those typically achieved during voluntary consumption by rodents or humans (≥50 mM), enhancement of GC GABA_A_R transmission occurs at low, readily achieved concentrations of alcohol (5–30 mM). Furthermore, although the changes in tonic GABA_A_R currents that are induced by such low concentrations of EtOH are small (1–5 pA), because of their constant nature, tonic GABA_A_R currents make up 75% of the total inhibitory current in GCs (Rossi and Hamann, 1998; Hamann et al., 2002a), which combined with the electrically compact nature of GCs (Rossi and Slater, 1993), enables such small tonic currents to profoundly influence transmission through the cerebellar cortex (Hamann et al., 2002a; Duguid et al., 2012). Thus, to the extent that EtOH actions at GABA_A_Rs influence acute EtOH sensitivity or voluntary consumption levels (Belelli and Lambert, 2005; Criswell et al., 2008; Kumar et al., 2009), the distinctively high sensitivity of GC GABA_A_R transmission makes it a primary candidate mechanism. Indeed, a growing body of research indicates that genetic differences in cerebellar processing and cerebellar responses to alcohol contribute to susceptibility to alcohol use disorders (AUDs) (Schuckit et al., 1996, 2005; Hill et al., 2007; Hill, 2010; Herting et al., 2011; Cservenka and Nagel, 2012), but the mechanisms by which the cerebellum influences the development of AUDs are not known.

Insight into cerebellar contributions to AUD risk comes from studies of the low level of response (LLR) to EtOH phenotype, which is defined as requiring a higher dose of EtOH to achieve a given effect. EtOH-induced static ataxia (body sway), a form of cerebellar-dependent motor impairment, consistently shows LLR in individuals with a family history of AUDs compared to individuals without a family history of AUDs (Schuckit et al., 1996, 2005). Thus, low cerebellar sensitivity to EtOH may be a risk factor for AUDs. In support of this contention, the magnitude of EtOH-induced ataxia shows an inverse relationship with EtOH consumption and preference in some inbred strains of mice (Gallaher et al., 1996; Yoneyama et al., 2008) as well as lines of rodents selected for differences in alcohol consumption (Malila, 1978; Bell et al., 2001, 2006) or in EtOH-induced motor impairment (McClearn et al., 1981; Sarviharju and Korpi, 1993). Importantly, cerebellar specific injections of various drugs can inhibit systemically administered EtOH induced-ataxia (Dar, 1995, 2011; Al Rejaie and Dar, 2006), clearly indicating the central role of the cerebellum in mediating EtOH-induced ataxia. Prompted by this consistent genetic relationship between cerebellar sensitivity to EtOH and AUDs in humans and excessive EtOH consumption in rodents, we recently examined GC tonic GABA_A_R current responses to EtOH in a range of rodent genotypes with divergent EtOH related phenotypes, and we found that the magnitude and polarity of the response varied, in a graded fashion, from strong enhancement in high sensitivity, low EtOH consuming rodents to suppression in low sensitivity, high EtOH consuming rodents (Kaplan et al., 2013). Thus, the consistent genetic relationship between EtOH consumption levels and cerebellar-dependent behavioral sensitivity to EtOH is reflected at a cellular level by genetic differences in GC tonic GABA_A_R current responses to EtOH.

There are several important but neglected issues in translating the abundant rodent literature to humans. First, it is possible that phylogenetic differences fundamentally alter the sites of action or impact of alcohol. For example, tonic GABA_A_R inhibition is known to be strongly regulated by astrocytic GABA transporters (Rossi et al., 2003), and the density of astrocytic processes relative to neuronal processes increases with phylogenetic advancement (Oberheim et al., 2006). In fact, it is not known if primate neurons exhibit tonic GABA_A_R mediated inhibition, or if they do, whether they are sensitive to EtOH. Indeed, we recently determined that systemic injections of EtOH (1–2 mg/kg) significantly increase serum levels of the GABA_A_R enhancing neurosteroids, pregenenolone and THDOC, in SDRs but not in NHPs (Porcu et al., 2010). Thus, much of what has been learned about cellular actions of EtOH in rodents may not translate to primates. Finally, in the context of genetic variation in cerebellar sensitivity to EtOH as a contributing factor to AUDs, most cellular studies have been conducted on genetically homogenous SDRs, who are very low EtOH consumers (i.e., under free access paradigms, they typically only consume 1–2 g/kg/day) (Duncan and Deitrich, 1980). Accordingly, the well documented EtOH enhancement of GC tonic GABA_A_R currents in SDR GCs may be the cellular phenotype of low risk for excessive EtOH consumption. Indeed, our recent findings that EtOH enhances GC tonic GABA_A_R currents in low EtOH consuming rodent genotypes but suppresses GC tonic GABA_A_R currents in high EtOH consuming rodent genotypes (Kaplan et al., 2013) raises the translationally important question: what is the response in NHPs, which on average show an intermediate EtOH consumption phenotype, and, being a genetically heterogenous population, show a wide range of consumption phenotypes across individual NHPs?

To address these issues, we conducted voltage-clamp recordings from GCs in cerebellar slices obtained from the NHP, *Macaca fascicularis*. We found that similar to SDRs, NHP GCs exhibit a tonic conductance that is generated by α6δ subunit containing GABA_A_ receptors. However, in contrast to SDR GCs, in most cells (60%), acute application of EtOH (25–105 mM) did not increase sIPSC frequency or the amplitude of the tonic GABA_A_R current. In a minority of cells (40%), EtOH did cause an increase in sIPSC frequency that was accompanied by a small increase in the amplitude of the tonic GABA_A_R current. Importantly, the proportion of cells that responded to EtOH varied considerably across individual NHPs, as does EtOH consumption. Thus, in contrast to genetically homogeneous, low EtOH consuming SDRs, in genetically diverse NHPs, EtOH-induced enhancement of GABA_A_R tonic currents is neither widespread nor homogenous across individuals, making it a potential contributing factor to genetic variation in sensitivity to and consumption of EtOH.

## Results

### NHP GCs exhibit phasic and tonic GABA_A_R currents indistinguishable from SDR GCs

**Figure 1:**
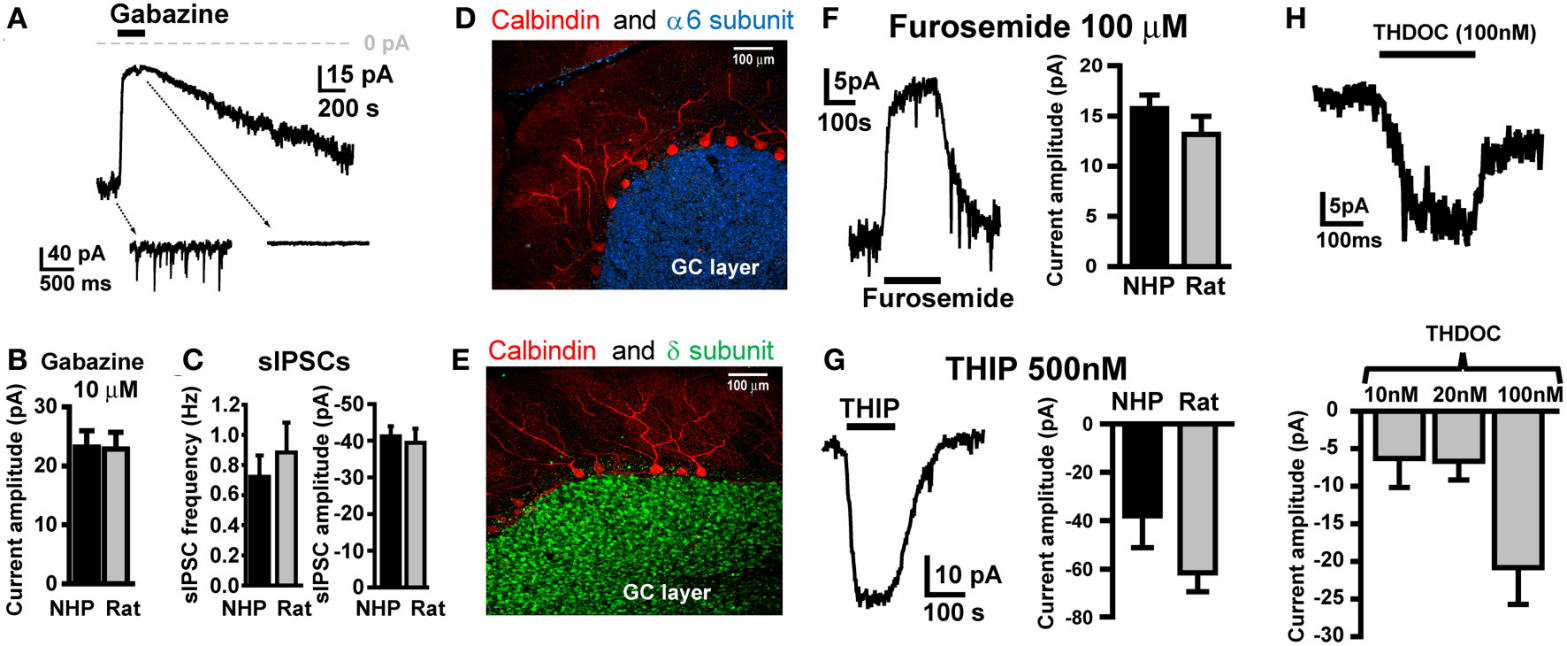
**NHP and SDR GCs exhibit similar magnitude tonic currents mediated by α6 and δ subunit containing GABA_A_Rs**. **(A)** Example trace showing block of tonic current and sIPSCs by GABA_A_R antagonist GABAzine (10 μM) in GCs from NHPs. Note, in this and all other figures, the dashed arrows point to an expanded time scale of recording from different time points of the main traces (coming from the region of the main trace that the back of the arrow extrapolates to), showing sIPSCs and block by GABAzine. **(B)** Plot summarizing mean amplitude of tonic current blocked by GABAzine (10 μM) in NHP (black; *n* = 37 GCs from 10 animals) and SDR (gray; *n* = 16 GCs from 7 animals) GCs. **(C)** Plot of mean frequency (left) and amplitude of sIPSCs (right) in NHP (black; *n* = 71 GCs from 12 animals) and SDR (gray; *n* = 64 GCs from 26 animals) GCs (**D,E)**. Confocally acquired fluorescence images of cerebellar slices from an NHP showing α6 (blue stain in **D**) and δ (green stain in **E**) subunits of the GABA_A_R expression exclusively in the granule cell layer (beneath the Purkinje cell layer, labeled red with antibody to Calbindin). **(F)** Example trace (left panel) showing block of tonic current by GABA_A_R antagonist furosemide (100μM, at which concentration it is specific for GABA_A_Rs containing the α6 subunit) in a GC from an NHP. Plot in right panel summarizes mean amplitude of tonic current blocked by furosemide in NHP (black; *n* = 53 GCs from 11 animals) and SDR (gray; *n* = 23 GCs from eight animals) GCs. **(G)** Example trace (left panel) showing enhancement of tonic current by GABA_A_R agonist THIP (500 nM, at which concentration it is specific for GABA_A_Rs containing the δ subunit) in a GC from an NHP. Plot in right panel summarizes mean amplitude of tonic current induced by THIP in NHP (black; *n* = 5GCs from two animals) and SDR (gray; *n* = 31 GCs from six animals) GCs. **(H)** Example trace (top panel) showing enhancement of tonic current by GABA_A_R modulator THDOC (100 nM) in a GC from an NHP. Plot in bottom panel summarizes mean amplitude of tonic current induced by 10, 20, and 100 nM THDOC in NHP GCs (*n* = 4, 6, and 3, respectively from two animals). Furosemide-, THIP-, GABAzine-, and THDOC-induced currents are all significantly different from zero *P* < 0.05, and none are significantly different between species *P >* 0.05 (excluding THDOC which was not examined in SDRs).

To characterize GABA_A_R-mediated inhibition in NHP GCs, we conducted voltage-clamp recordings from GCs in thin cerebellar slices. In all recordings, GCs were clamped at −60 mV, with E*_Cl_*- set to near 0 mV, so all GABA_A_R-mediated currents are inward (downward deflections of holding current traces), and conversely, blockade of GABA_A_R currents will result in outward currents (upward deflections). Similar to what we and others have reported for SDR GCs (Brickley et al., 1996; Wall and Usowicz, 1997; Hamann et al., 2002a), NHP GCs exhibited tonic GABA_A_R currents with superimposed phasic GABA_A_R-mediated sIPSCs, both of which were blocked by the broad spectrum GABA_A_R antagonist, GABAzine (10 μM; Figure 1A). The magnitude of the tonic GABA_A_R current, and the frequency and magnitude of sIPSCs were nearly identical in NHP and SDR GCs (Figures 1B,C; *P* > 0.05 for all, *n* = 37 NHP GCs from 10 animals and 16 SDR GCs from 7 animals). We previously determined that in SDR GCs, the tonic current is mediated by extrasynaptically located GABA_A_Rs containing α6 and δ subunits, which endow the receptor with properties optimized for sensing low ambient concentrations of extracellular GABA (Brickley et al., 2001; Hamann et al., 2002a; Stell et al., 2003). Accordingly, we used immunocytochemistry and confocal fluorescence microscopy to determine if NHP GCs expressed α6 and δ subunits (Figures 1D,E). Similar to the SDR cerebellum, both the α6 and δ subunit are densely and selectively expressed in a confluent pattern across the GC layer, presumably, based on their numerical dominance, on GCs (Figures 1D,E). Next, we used subunit selective ligands to confirm that NHP GC tonic GABA_A_R currents were indeed mediated by GABA_A_Rs containing α6 and δ subunits (Figures 1F,G). The tonic GABA_A_R current was reduced by furosemide [100 μM, at which concentration it is specific for GABA_A_Rs containing the α6 subunit (Korpi et al., 1995; Hamann et al., 2002a), Figure 1F], and was enhanced by the GABA_A_R agonist THIP [500nM, which at concentrations up to 1 μM is specific for GABA_A_Rs containing δ subunits (Meera et al., 2011), Figure 1G]. Finally, NHP GC tonic GABA_A_R currents were also enhanced by low, physiologically relevant concentrations of the neurosteroid THDOC (Figure 1H). These data confirm that, similar to previous reports in SDR GCs, NHP GCs exhibit tonic GABA_A_R currents mediated by extrasynaptic α6δ-containing receptors (Hamann et al., 2002a). There were no detectable differences between NHP and SDR GC GABA_A_R mediated sIPSCs or tonic current (Figures 1B,C,F,G).

### NHP GCs exhibit a spillover component to electrically-evoked GABA_A_R IPSCs

**Figure 2:**
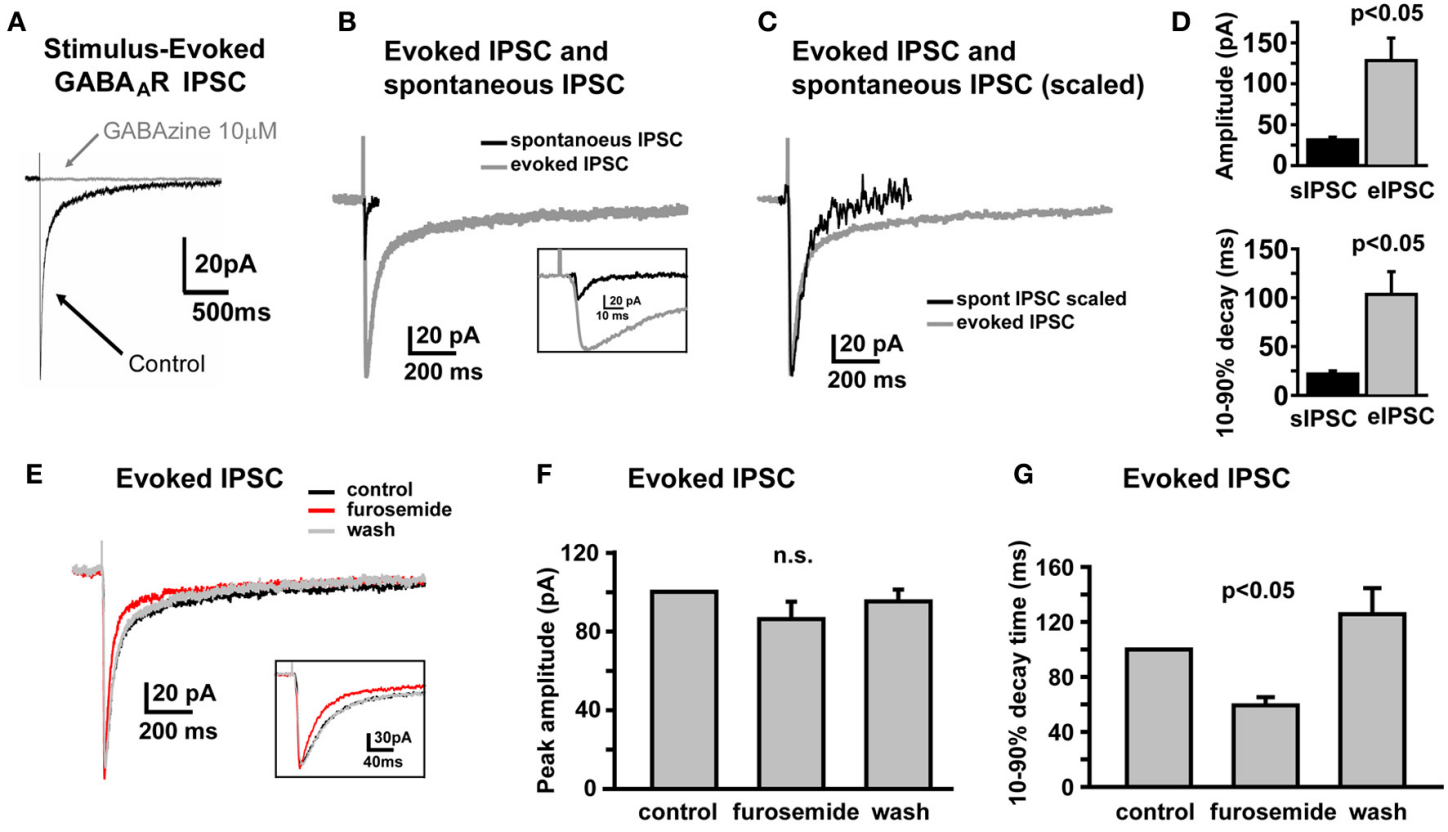
**Electrically evoked IPSCs exhibit a prolonged tail component mediated by GABA_A_Rs containing the α6 subunit. (A)** Example trace showing that an electrically-evoked synaptic current, that is abolished by the GABA_A_R antagonist, GABAzine (10 μM), confirming that it was a GABA_A_R-mediated IPSC. **(B,C)** Representative traces showing the sIPSC (black) and eIPSC (gray) from the same NHP GC, overlaid at actual size **(B)** and scaled to peak **(C)** to illustrate prolonged decay time of eIPSC relative to the sIPSC in the same cell. Inset in **(B)** shows the early phase of sIPSC and eIPSC at an expanded time scale. **(D)** Plots showing mean amplitude (top) and 10–90% decay time (bottom) for sIPSCs and eIPSCs in the same NHP GCs, *n* = 6 GCs from two animals. **(E)** Representative trace showing that furosemide reduces the decay time of the eIPSC without affecting the peak amplitude. Inset shows the early phase of the eIPSC at an expanded time scale. **(F,G)** Plots of the mean impact of furosemide (and recovery after wash) on the peak amplitude **(F)** and 10–90% decay time **(G)** of eIPSCs in NHP GCs, normalized to the control values, *n* = 4 GCs from two animals.

In SDR GCs, one apparent role of high affinity α6 containing GABA_A_Rs is to respond to spillover of GABA from neighboring active synapses that do not form direct synapses with the recoded GC (Rossi and Hamann, 1998). In SDRs, this spillover response manifests as a prolonged tail component to GABA_A_R IPSCs that are evoked by electrical stimulation, which by its nature tends to activate both connected synapses and neighboring but unconnected synaptic afferents simultaneously. Similar to SDR GCs, electrical stimulation of NHP GC afferents evoked a GABA_A_R-mediated, phasic IPSC that was abolished by the broad spectrum GABA_A_R antagonist, GABAzine (10 μM, Figure 2A, *n* = 7). As for SDR GCs, the electrically evoked IPSC (eIPSC) in NHP GCs was significantly larger, and decayed much more slowly than sIPSCs (Figures 2B–D, *n* = 4, *P* < 0.05 for both amplitude and decay time). Furthermore, the decay time, but not the amplitude of the eIPSC was significantly (*P* < 0.05) reduced by the α6 subunit specific antagonist, furosemide (100 μM; Figures 2E–G, *n* = 4). Thus, similar to SDR GCs, NHP GCs respond to transient elevations of extracellular GABA induced by activation of multiple neighboring but not directly connected synapses.

### EtOH does not affect GABA_A_R currents in the majority of NHP GCs

**Figure 3:**
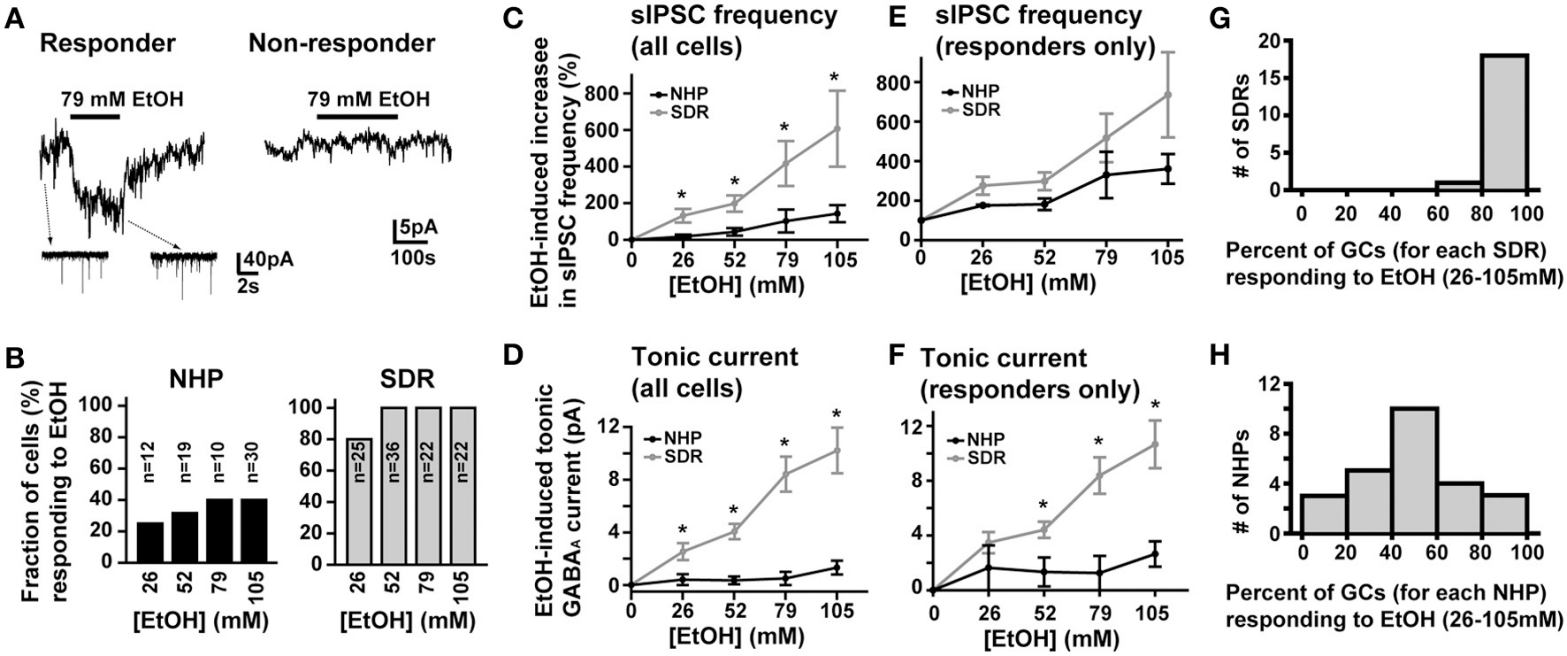
**EtOH enhancement of GC sIPSC frequency and tonic GABA_A_R current magnitude is restricted and variable in NHPs. (A)** Representative data showing that EtOH can either increase sIPSC frequency (insets) and potentiate the tonic GABA_A_R current magnitude (main trace left panel) or have no impact on tonic GABA_A_R currents (right panel) or sIPSC frequency (not shown) in NHP GCs. **(B)** Plot of proportion of GCs showing potentiation or no effect of EtOH on tonic GABA_A_R currents and sIPSC frequency in SDR (right, gray) and NHP (left, black) GCs. **(C,E)** Plot of the mean percent increase in sIPSC frequency induced by EtOH for all cells tested **(C)** or EtOH responding cells only **(E)** from SDRs (gray) and NHPs (black) [^*^ indicates significantly different, *P* < 0.05, *n* = 21–35 GCs from 9–20 animals for each dose of EtOH, see panel **(B)** for details of proportion of responders and non-responders]. **(D,F)** Plot of the mean magnitude of the GC GABA_A_R tonic current induced by EtOH for all cells tested **(D)** or EtOH responding cells only **(F)** from SDRs (gray) and NHPs (black) [^*^ indicates significantly different, *P* < 0.05, *n* = 21–35 GCs from 9–20 animals for each dose of EtOH, see panel **(B)** for details of proportion of responders and non-responders]. Note, in all responding cells examined, subsequent application of GABAzine (10 μM) abolished all EtOH-induced currents. **(G,H)** Plot of the proportion of GCs from individual animals that responded to EtOH at any dose of EtOH tested. The distribution for SDRs and NHPs are plotted in **(G)** and **(H)** respectively.

**Figure 4:**
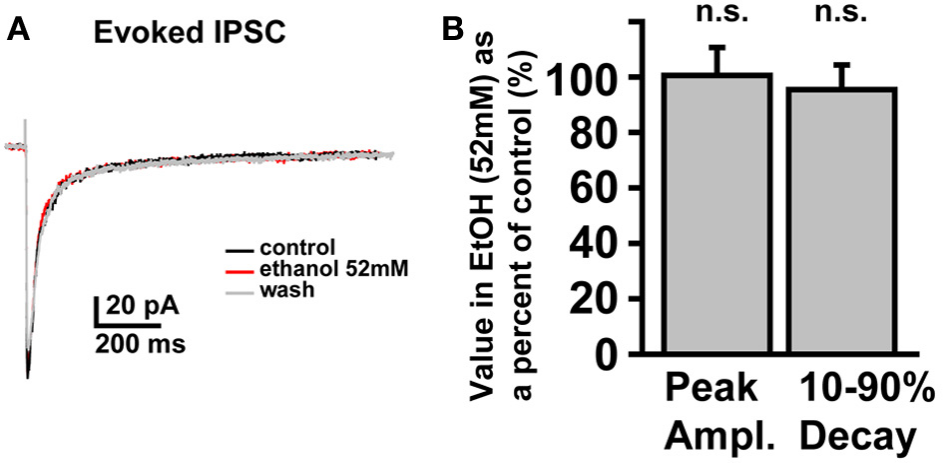
**Electrically evoked IPSCs are not affected by EtOH**. **(A)** Example trace showing that an eIPSC in an NHP GC is not affected by EtOH (52 mM). **(B)** Plot of the mean percent change, induced by EtOH (52 mM), of the peak amplitude (left bar) and 10–90% decay time (right bar) of eIPSCs in NHP GCs, normalized to the control values (before and after EtOH), *n* = 4 GCs from two animals.

In SDRs, the GC GABA_A_R system is a well-documented primary target of physiologically relevant concentrations of EtOH (5–30 mM) (Carta et al., 2004; Botta et al., 2007b; Olsen et al., 2007), with EtOH-induced enhancement of GC sIPSC frequency and tonic GABA_A_R current magnitude likely mediating many aspects of acute intoxication and impairment. Surprisingly, although a similar enhancement by EtOH was observed in some NHP GCs (~40%: 3 of 12 at 26 mM, 6 of 19 at 52 mM, 4 of 10 at 79 mM, 12 of 30 at 105 mM), in the majority of NHP GCs (~60%: 9 of 12 at 26 mM, 13 of 19 at 52 mM, 6 of 10 at 79 mM, 18 of 30 at 105 mM) EtOH did not affect sIPSC frequency or the magnitude of tonic GABA_A_R currents (Figures 3A,B). Accordingly, the mean percent increase in sIPSC frequency and tonic GABA_A_R current magnitude, averaged across NHP GCs, was significantly smaller than SDR GCs at all doses of EtOH examined (Figures 3C,D). If we excluded NHP GCs that did not respond to EtOH, and plotted the mean impact of EtOH on NHP GCs that did respond to EtOH, the impact on sIPSCs was similar between NHP and SDR GCs, but the impact on the tonic GABA_A_R current was still significantly reduced in NHP GCs (Figures 3E,F). Interestingly, the proportion of cells that responded to EtOH was quite variable from animal to animal, ranging from 0 to 100% responders (Figures 3G,H), as would be expected in a genetically heterogeneous population, if the cellular response phenotype contributes to genetic differences in EtOH related behaviors. As a further test of NHP GC GABA_A_R sensitivity to EtOH, we also determined that high concentrations of EtOH (52 mM) also did not affect the amplitude of eIPSCs, or the decay rate of the spillover component (Figure 4). We did not examine EtOH actions on SDR evoked IPSCs, since there were so few non-responders to examine.

### Lack of response to EtOH is not due to lack of functional afferents, or differences in GABA_A_R receptor or GABA transporter density

**Figure 5:**
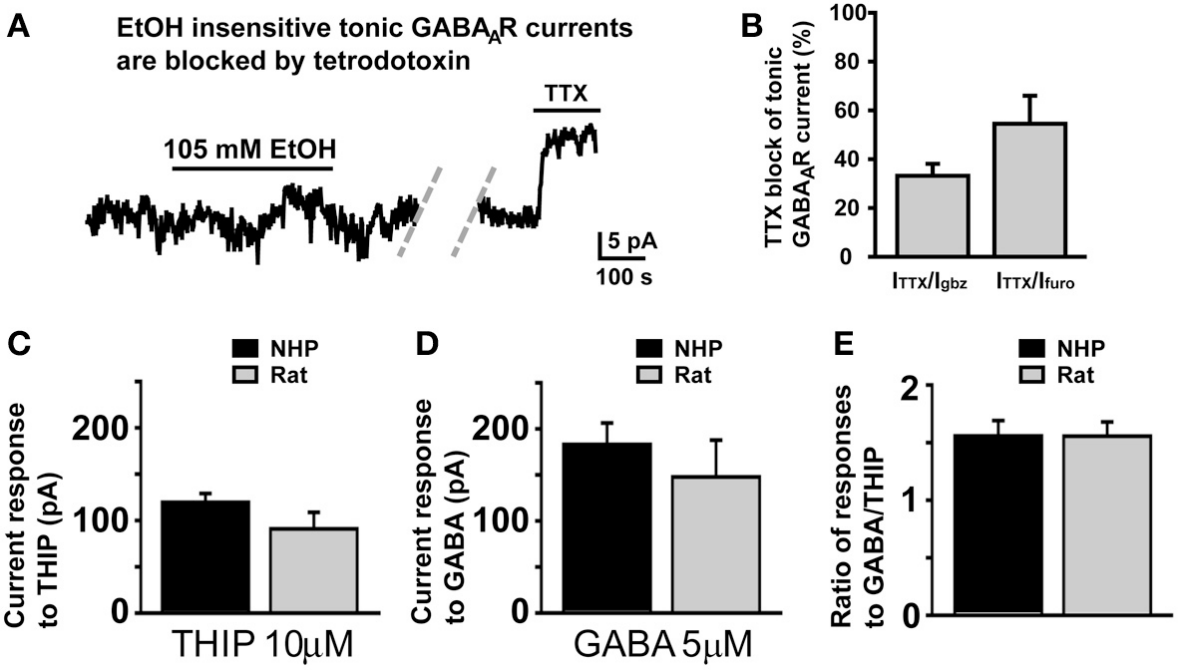
**Lack of response to EtOH is not due to damaged Golgi cells or differences in GABA_A_R or GABA transporter density**. **(A)** Representative trace showing that in an NHP GC that did not respond to EtOH (105 mM), subsequent application of TTX (500 nM) significantly reduced the magnitude of the tonic GABA_A_R current. Dashed doublet of gray diagonal lines indicates a gap in the current shown to conserve figure space. **(B)** Plot of mean percent block by TTX (500 nM) of tonic GABA_A_R current, expressed as the percent of the tonic GABA_A_R current blocked by the broad spectrum GABA_A_R antagonist, GABAzine (10 μM, left bar) or the α6 specific antagonist, furosemide (100 μM, right bar), *n* = 7 and 6 GCs respectively from two animals. **(C–E)** Plots of mean current induced by THIP (10 μM, **C**), GABA (5 μM, **D**), and the ratio of the amplitudes of those responses in individual GCs **(E)**. For each plot, data from NHPs and SDRs are plotted in black and gray respectively (*n* = 4–10 GCs each from 2 to 3 animals).

Since in SDRs, the majority (Hanchar et al., 2005), if not all (Botta et al., 2007a,b), of EtOH-induced increases in tonic GABA_A_R currents is a consequence of increased extracellular GABA, via increased vesicular release, we considered that the lack of effect of EtOH in NHP GCs might result from a loss of the GC GABA afferent Golgi cells due to damage during slicing. However, in 4 NHP GCs that did not respond to EtOH (105 mM), subsequent application of the sodium channel blocker, TTX (500 nM), strongly suppressed the frequency of sIPSCs (not shown; sIPSC frequency = 0.66 ± 0.34 and 0.04 ± 0.02 Hz before and after TTX, respectively; *P* < 0.02) and the magnitude of the tonic GABA_A_R current (Figures 5A,B). Indeed, the TTX-induced suppression of tonic GABA_A_R currents in non-responding NHP GCs was significantly greater than TTX-induced suppression of tonic GABA_A_R currents in SDR GCs (TTX-induced current was 12.05 ± 1.27 and 4.35 ± 0.60 pA in non-responding NHP and SDR GCs respectively, which corresponded to 38.45 ± 4.05 and 16.18 ± 2.96%, respectively, of the GABAzine-induced current in the same cell; *P* < 0.001 for both measures), suggesting that vesicular release of GABA from healthy Golgi cells plays a more prominent role in regulating GABA_A_R currents in NHP GCs (even non-responding NHP GCs) than in SDR GCs. Thus, the lack of enhancement by EtOH is not due to lack of action potential competent Golgi cells, able to modulate the extracellular GABA around the recorded GC.

We next considered that if NHP GCs had a lower density of GABA_A_Rs, then EtOH-induced GABA release at neighboring synapses may not generate large enough currents to be detected in NHP GCs. However, bath application of a concentration of THIP (10 μM) or GABA (5 μM) that should activate all subtypes of GABA_A_Rs produced similar magnitude currents in NHP and SDR GCs (Figure 5C), suggesting similar densities of all subtypes of GABA_A_Rs, similar to what we observed for the subtypes that mediate tonic GABA_A_R currents under control conditions, which are also of similar density (Figures 1F,G). Another possibility we considered was that higher densities of GABA transporters in NHPs might reduce EtOH-induced GABA spillover and/or accumulation in the extracellular space, thereby preventing enhancement of tonic GABA_A_R currents. To assess GABA transporter functional density, we used a technique that we have used previously to assess both glutamate (Hamann et al., 2002b, 2005) and GABA (Rossi et al., 2003; Allen et al., 2004) transporter densities, whereby we do cell by cell comparisons of the ratio of response to GABA, which is taken up by GABA transporters, and THIP which is not taken up by GABA transporters. Since the density of GABA transporters will only influence the concentration of GABA in the extracellular space, and will not affect the concentration of THIP, then differences in GABA transporter densities will change the ratio of response to the two GABA_A_R agonists. However, there was no significant difference in the ratio of responses to THIP and GABA between NHP and SDR GCs (Figure 5E), suggesting similar functional densities of GABA transporters. Taken together, our data indicate that GABA_A_R pharmacology and physiology, GABA transporter functional density, and Golgi cell viability are similar between NHPs and SDRs, and thus the lack of impact of EtOH on sIPSC frequency and tonic GABA_A_R current in NHP GCs is due to a lack of impact on Golgi cell firing.

In addition to comparing NHP GCs to SDR GCs, we considered whether there were any obvious differences between EtOH-responding and non-responding NHPs. Although the magnitude of the tonic GABA_A_R current was similar in responding and non-responding NHP GCs (Furosemide-induced current = 15.8 ± 2.19 and 21.39 ± 3.05 pA in responding and non-responding NHP GCs, respectively; GABAzine-induced current = 23.65 ± 5.78 and 27.13 ± 6.73 pA in responding and non-responding NHP GCs respectively; *P* > 0.05 for both), the baseline frequency of sIPSCs was significantly lower in non-responding NHP GCs (0.61 ± 0.16 and 0.29 ± 0.11 Hz in responding and non-responding NHP GCs respectively; *P* < 0.05). These data suggest that Golgi cells that form direct synaptic contacts with non-responding NHP GCs are either fewer in number or fire action potentials less frequently, but that the ambient concentration of GABA surrounding EtOH non-responding NHP GCs is similar to that around responding NHP GCs.

### Reduced and variable response to EtOH is mirrored by reduced and variable expression of nNOS

**Figure 6:**
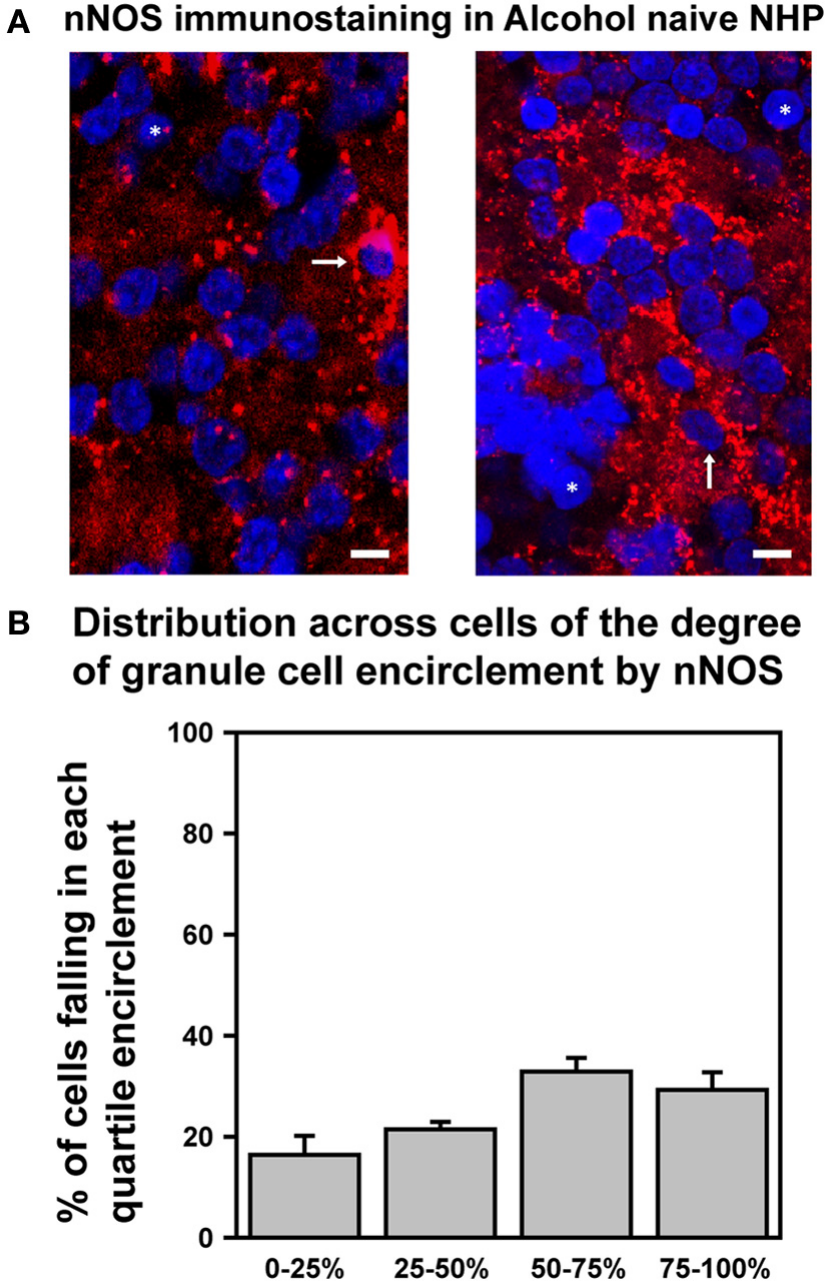
**Differential expression of nNOS mirrors differences in EtOH-induced potentiation of GC GABA_A_R transmission. (A)** Confocally acquired images of immunocytochemistry for nNOS (red) and nuclear stain Hoechst (blue) in the granule cell layer of NHP cerebellum. Arrows point to examples of GC somas completely surrounded by nNOS signal, and ^*^ indicate examples of GC somas completely devoid of nNOS encirclement. **(B)** Plot of mean percent encirclement of GC soma/nuclei (determined as described in Kaplan et al. (2013); *n* = 2,569 GCs from six animals).

We recently determined that in SDRs, EtOH-induced enhancement of GC tonic GABA_A_R currents is triggered by EtOH suppression of neuronal nitric oxide synthase (nNOS), which in turn drives increased Golgi cell action potential firing and consequent increased vesicular release of GABA (Kaplan et al., 2013). We also found that prototypically high EtOH consuming C57BL/6J (B6) mice had significantly lower levels of expression of nNOS in the GC layer, and accordingly that EtOH enhancement of GC GABA_A_R current only occurred in 15% of B6 GCs (Kaplan et al., 2013). We therefore reasoned that the relative lack of response of NHP GC tonic GABA_A_R currents was due to a similarly reduced expression of nNOS. To test this hypothesis, we used immunocytochemistry and confocal microscopy to examine nNOS expression in the GC layer of the NHP cerebellum (Figure 6). Although we did detect nNOS expression in the NHP cerebellum, and the subcellular localization pattern was qualitatively similar to what we reported for SDRs (i.e., it was frequently concentrated in a halo surrounding GC nuclei; Figure 6A), in support of our hypothesis, the presence of nNOS expression around GC nuclei was more variable, and generally less prevalent than what we reported for SDRs (Figures 6A,B) (Kaplan et al., 2013). In particular, while some NHP GCs were entirely surrounded by nNOS [similar to the majority of SDR GCs (Kaplan et al., 2013)], the majority of NHP GCs were not fully surrounded by nNOS, and many NHP GCs were entirely devoid of nNOS. These data, combined with our recent studies in several rodent genotypes with divergent EtOH related phenotypes, suggest that the relatively reduced and more varied response of NHP GCs to EtOH stems from reduced and more varied expression of nNOS.

## Discussion

Using voltage-clamp recording from GCs in acutely prepared slices of NHP cerebellum, we have determined that GABA_A_R-mediated transmission onto NHP GCs is physiologically and pharmacologically indistinguishable from SDR GCs (Figures 1, 2, 5). In particular, the frequency and amplitude of sIPSCs and the magnitude of tonic GABA_A_R currents are similar, the response of the tonic GABA_A_R current to various subunit specific ligands is similar, and the density of GABA_A_R receptors and subunits, and the density of GABA transporters are similar. Thus, despite potential differences in the ratio of astrocytes and neurons across phylogenetic advancement (Oberheim et al., 2006), the NHP GC synapse functions similarly to SDR GCs, in that they both exhibit traditional phasic synaptic IPSCs and sense spillover of GABA from neighboring unconnected synapses as well as the ambient concentration of GABA in the extracellular space. Despite nearly identical baseline properties, in striking contrast to SDR GCs (Carta et al., 2004; Hanchar et al., 2005), in the majority of NHP GCs, EtOH does not affect GABA_A_R mediated transmission, even at extremely high, non-physiological concentrations (Figure 3).

### Mechanisms enabling GC responses to ambient and spillover GABA

In SDRs, the ability of GCs to sense spillover and ambient GABA is thought to be achieved through a combination of receptor pharmacology and subcellular morphological interactions (Rossi and Hamann, 1998; Brickley et al., 2001; Hamann et al., 2002a; Stell et al., 2003). In particular, inclusion of the α6 and δ subunits into functional GABA_A_Rs endow the receptor with an order of magnitude higher affinity for GABA than other GABA_A_Rs (Saxena and Macdonald, 1996), enabling them to sense the low concentrations of ambient GABA that occur in the extracellular space and during spillover from neighboring synapses (Rossi and Hamann, 1998; Hamann et al., 2002a). Further facilitation is achieved by the glomerular structure in which Golgi cell to GC synapses occur (Rossi and Hamann, 1998). The glomerulus is comprised of approximately 50 neighboring GC synapses in close proximity, and rather than being separated by astrocytic processes as occurs in the forebrain, they are collectively encased by an astrocytic sheath (Jakab and Hamori, 1988), thereby facilitating transmitter cross talk among intraglomerular synapses, and retention of ambient transmitter in the local extracellular space (Rossi and Hamann, 1998).

Using immunohistochemistry, we determined that similar to SDRs, both the α6 and δ subunit of the GABA_A_R are densely and selectively expressed by NHP GCs (Figures 1D,E). Furthermore, using α6 and δ subunit selective ligands, we confirmed that the tonic GABA_A_R is mediated by GABA_A_Rs containing both of these subunits, and that the spillover component of the eIPSC is mediated by GABA_A_Rs containing at least the α6 subunit (Figure 2). Because we primarily test for the presence of the δ subunit with the selective agonist, THIP, and interpreting actions of agonists on synaptic decay rates is complicated, we were unable to test for the contribution of δ subunits to the spillover component. Taken together our immunohistochemical and pharmacological studies suggest that similar to SDRs, NHP GCs express both the α6 and δ subunits of the GABA_A_R, and that their presence in functional GABA_A_Rs enables NHP GCs to sense ambient and spillover GABA.

To our knowledge a detailed 3-dimensional reconstruction of electron micrographs of the GC layer has only been done in the rat (Jakab and Hamori, 1988). However, an earlier study using light microscopic examination of Golgi preparations combined with single plane electron microscopy suggest that the primary features of the glomerulus in the NHP is similar to that in SDRs, including an astrocytic sheath around multiple GC dendrites (Fox et al., 1967). Thus, the molecular, pharmacological, and anatomical characteristics of the Golgi cell to GC synapse appear to be relatively conserved across species, resulting in remarkably similar physiological characteristics (Figures 1, 2).

### EtOH does not affect GABA_A_R currents in the majority of NHP GCs

It is well established that the SDR GC GABA_A_R system is highly sensitive to EtOH, with concentrations as low as 10 mM (i.e., the equivalent of those achieved by an adult human consuming ~2 units of alcohol) substantially increasing sIPSC frequency and tonic GABA_A_R current magnitude (Carta et al., 2004; Hanchar et al., 2005). Furthermore, because of the electrically compact nature of GCs, their central role in signal propagation through the cerebellar cortex, and the dominant role of tonic GABA_A_R currents in mediating GC GABA_A_R inhibition, even small alterations of the tonic GABA_A_R current powerfully influence signal propagation through the cerebellar cortex (Hamann et al., 2002a; Duguid et al., 2012). Thus, even low concentrations of EtOH significantly disrupt SDR cerebellar processing (Hanchar et al., 2005). In contrast, other than the central nucleus of the amygdala (Roberto et al., 2003), the GABA_A_R system in most other brain nuclei (e.g., hippocampus, nucleus accumbens, thalamus, ventral tegmental area, and substantia nigra; Peris et al., 1992; Nie et al., 2000; Liang et al., 2006; Jia et al., 2008; Theile et al., 2008, 2009), is not affected by concentrations of EtOH below ~50 mM EtOH. Given that even the most avid rodent consumers of EtOH (e.g., high drinking in the dark selected mice, C57BL/6 mice and alcohol preferring rats) only voluntarily consume enough EtOH to reach BECs of ~20–25 mM, it is likely that in rodents, the GC GABA_A_R system is a primary mediator of symptoms of acute intoxication. However, we found that in the majority (~60%) of NHP GCs, even extremely high concentrations of EtOH (105 mM) do not affect any aspect of GABA_A_R transmission (Figure 3). The lack of response to EtOH was not due to a lack of functional Golgi cell afferents, because blocking action potentials with TTX substantially reduced the frequency of sIPSCs and the magnitude of the tonic GABA_A_R current (Figures 5A,B), clearly indicating that functional Golgi cells were present, connected, and able to influence GC GABA_A_R currents. The lack of response to EtOH also did not reflect differences in the density of GABA_A_Rs or GABA transporters, as pharmacological assessment of these parameters found no significant differences from SDRs (Figures 5C–E). Thus, the most parsimonious explanation for the lack of response to EtOH is that in contrast to SDRs, the majority of NHP Golgi cells do not respond to EtOH with an increase in action potential firing, and thus there is no increase in vesicular GABA release, which normally mediates EtOH-induced increases in both sIPSC frequency and the tonic GABA_A_R current magnitude (Carta et al., 2004; Botta et al., 2007a,b, 2010, 2012). Indeed, the relative lack of, and highly variable response of NHP GCs to EtOH is mirrored by reduced and highly variable cerebellar expression of nNOS (Figure 6), the suppression of which mediates EtOH-induced excitation of Golgi cells and consequent enhancement of GC GABA_A_R currents in SDRs (Kaplan et al., 2013).

Although we did not systematically examine whether direct interactions of EtOH with GABA_A_Rs contribute to the EtOH-induced enhancement of tonic GABA_A_R currents, the fact that EtOH did not affect the tonic current at all in ~60% of cells (Figure 3B), and did not affect the peak or spillover component of eIPSCs (Figure 4), suggests that even high concentrations of EtOH do not directly affect NHP GC GABA_A_R sensitivity to GABA (Borghese et al., 2006; Botta et al., 2007a,b; Korpi et al., 2007; Olsen et al., 2007). Instead, when EtOH does affect NHP GC GABA_A_R transmission it is via excitation of Golgi cells and consequent increased vesicular release of GABA, similar to what has been reported for SDRs (Carta et al., 2004; Botta et al., 2007a,b, 2010, 2012).

In NHP GCs that did respond to EtOH, similar to SDR GCs, the response consisted of an increase in sIPSC frequency and an associated increase in the magnitude of the tonic GABA_A_R current (Figures 3A–D). However, although the increase in sIPSCs was similar to SDR increases, the magnitude of the EtOH-induced increase in the tonic GABA_A_R current was still significantly less than what occurred in SDR GCs (Figures 3E,F). This suggests that despite being connected to an EtOH-responding Golgi cell, neighboring, not directly connected Golgi cells did not respond to EtOH, resulting in less spillover and accumulation of GABA in the extracellular space, compared to SDRs, in which all Golgi cells respond to EtOH.

Responding NHP GCs responded to concentrations of EtOH as low as 25 mM, but because the currents are small and uncommon, combined with restricted access to NHP tissue, we did not test lower concentrations. Taken together, our data suggest that the impact of EtOH on cerebellar processing in NHPs is likely to be less substantial and less widespread than in SDRs.

### Potential relevance to EtOH related behavioral phenotypes

In SDRs, EtOH-induced enhancement of GABA_A_R currents occurs in ~95% of GCs (Figure 3B). Accordingly, EtOH will essentially dampen signal transmission through GCs confluently across the SDR cerebellum. The impact of such widespread dampening on cerebellar output is not entirely certain, because although GCs provide glutamatergic excitation to Purkinje cells (the sole output of the cerebellar cortex), they also provide such excitation to molecular layer interneurons, which, in turn, inhibit Purkinje cells (Duguid et al., 2012; Cesana et al., 2013). Our previous slice studies determined that widespread suppression of GC GABA_A_R inhibition increased the input to output relationship of transmission through the cerebellar cortex (Hamann et al., 2002a), but slice studies inevitably alter the full spatiotemporal relationship of inhibitory and excitatory neurons that exist in the intact cerebellum. In any event, our finding that EtOH enhances GABA_A_R transmission in only ~40% of NHP GCs (Figure 3B) suggests that the impact of EtOH on signal transmission through the cerebellar cortex of NHPs will be reduced relative to SDRs. Furthermore, depending on the spatial distribution of cells that respond to EtOH, downstream interactions with molecular layer interneurons and Purkinje cells (Duguid et al., 2012; Cesana et al., 2013; Cramer et al., 2013) could result in EtOH having opposite, or at least varying impact on cerebellar output in different cerebellar sub regions. It is also worth noting that although NHP GC tonic GABA_A_R currents are highly sensitive to enhancement by the neuroactive steroid, THDOC (Figure 1H), our recent studies determined that in contrast to SDRs, systemic EtOH does not increase serum levels of GABA_A_R-active neurosteroids in NHPs (Porcu et al., 2010). This lack of increase in GABA_A_R-active steroids may further reduce cerebellar responses to systemic EtOH in NHPs relative to SDRs. In addition to acute actions of EtOH, the relative lack of response of NHP GC GABA_A_R currents to EtOH could underlie reported species differences in molecular adaptation during chronic EtOH consumption. In particular, in a variety of models of chronic EtOH exposure, rodent cerebellum exhibits changes in GABA_A_R subunit expression, including the α6 and δ subunits (up and down regulated, respectively) that mediate tonic GABA_A_R currents (Marutha Ravindran et al., 2007a,b). In contrast, we reported previously that the expression of the α6 subunit in the NHP cerebellum is not altered by chronic voluntary EtOH consumption (Sullivan et al., 2005). While such differences may simply reflect the higher levels of EtOH used in rodent chronic exposure models, it could also stem from the lack of acute response that we report here (i.e., if GABA_A_R adaptations are homeostatic, then the lack of acute response to EtOH in NHP GCs may preclude adaptation during chronic EtOH exposure).

Beyond differences between NHPs and SDRs, we discovered that the proportion of NHP GCs that responds to EtOH varies widely (from 0 to 100%) across individual NHPs (Figure 3H). This wide individual variation in sensitivity is intriguing because in the human literature, variations in sensitivity to EtOH-induced body sway (a presumed cerebellar dependent behavioral impact of EtOH) correlates with genetic risk for developing an AUD. In particular, a LLR to EtOH-induced body sway is significantly correlated with a family history of alcoholism and, in longitudinal studies, with the development of alcoholism (Schuckit et al., 1996, 2005). Variation in behavioral sensitivity must be driven by variation in sensitivity at a cellular/molecular level, but prior to our study of genetically heterogeneous NHPs, no such individual variation at a cellular level had been reported, presumably because most rodent models, whether high or low EtOH consumers, are genetically homogeneous. Thus, variation in the proportion of GCs that respond to EtOH could be a cellular substrate of the LLR to EtOH-induced body sway, and thus a potential cellular/molecular risk factor for AUDs. It would therefore be useful to identify the molecular determinants of EtOH-induced enhancement of GC GABA_A_R currents (Hanchar et al., 2005; Botta et al., 2007b, 2010, 2012; Diaz et al., 2011; Kaplan et al., 2013), and to determine whether manipulating GC GABA_A_R current responses to EtOH can influence EtOH consumption in rodent and NHP models.

### Limitations of this study

Access to NHP tissue is rare but valuable. Thus, while such access has the potential to provide translationally important insights not possible to glean from rodent studies, it does bring with it a variety of experimental limitations. In particular, we rely on control NHPs destined for euthanasia as part of other larger behavioral studies, which typically only provide about 4–8 useable subjects per year. Thus, while our study is not as thorough as would be possible with easily accessible rodents, the individual experiments are sound, and our findings should prompt an important reconsideration of the actions of alcohol in the clinical context, and should serve as an important stimulus and starting point for further future studies. Nonetheless, some potential limitations merit consideration. First, we conducted all experiments at room temperature (22–24°C), which could in principle affect responses to EtOH. We opted for room temperature recording because in our experience recordings tend to be more stable (which is particularly crucial when recording small modifications of small currents), and because heating solutions in a room temperature room increases degassing of the solution, which creates bubbles, which can lodge in tubing and/or dislodge, either of which can disrupt a recording. Furthermore, over the years, we have varied temperature to determine if doing so affects various outcomes, and although most responses are faster at higher temperatures, few if any observations are fundamentally altered by changes in temperature (e.g., Rossi and Hamann, 1998; Hamann et al., 2005). For these reasons, to optimize our success rate with very limited access to NHP tissue, we chose to record at room temperature. Nonetheless, it is conceivable that lowered temperature has a differential impact on responses in rodents compared to NHPs, and such a differential response could explain the differences between rat and NHP responses to EtOH that we report. However, our finding that nNOS expression in NHPs is reduced and more variable, relative to SDRs (Figure 6), combined with our recent finding that EtOH-induced enhancement of GABA_A_R sIPSCs and tonic GABA_A_R currents in SDRs and other rodent genotypes is mediated by EtOH inhibition of nNOS (Kaplan et al., 2013) provides a plausible alternative mechanism, i.e., the relative lack of response of NHP GCs to EtOH is likely due to the lack of nNOS expression. In this regard, another limitation of this study is that because of the aforementioned limited access to NHP tissue, we have not yet confirmed that similar to our findings in rodents (Kaplan et al., 2013), EtOH inhibits nNOS in primates, with a resultant excitation of Golgi cells and consequent increased vesicular release of GABA. Such an undertaking should be a priority in future studies of NHP GC responses to EtOH.

Although previous studies have established that there is a consistent genetic relationship between cerebellar-dependent behavioral sensitivity to EtOH and predilection to AUDs in humans and excessive EtOH consumption in rodents (Malila, 1978; McClearn et al., 1981; Gallaher et al., 1996; Schuckit et al., 1996, 2005; Bell et al., 2001, 2006; Sarviharju et al., 2006; Yoneyama et al., 2008), to our knowledge no such relationship between NHPs and rodents or between individual NHPs has been established, and it was beyond the scope of this study to establish such a relationship. However, our finding that NHP GC GABA_A_R current responses to EtOH are reduced and more varied compared to low EtOH consuming and genetically homogeneous SDRs (Figure 3), combined with our findings that EtOH response magnitude and polarity correlates with sensitivity to and consumption of EtOH in rodent models (Kaplan et al., 2013) highlights the GC GABA_A_R system as a target to manipulate, to test whether the sensitivity to or consumption of EtOH are influenced by GC responses to EtOH.

Finally, because of complications associated with repeated applications of EtOH and incomplete washout of various GABA_A_R ligands, we did not examine responses to EtOH in multiple cells within a slice, and thus did not determine the overall 3-dimensional spatial distribution of NHP GCs that responded to EtOH and those that did not respond to EtOH. Such information will be important for considering what the net impact of EtOH will be on signal transmission through the NHP cerebellar cortex. Does EtOH dampen signal transmission or signal to noise ratios (Duguid et al., 2012) in some regions but enhance it or not affect it in other regions? Does it enhance signaling contrast between individual cells or cerebellar subregions? Future mapping of EtOH response phenotype across cerebellar subregions, and perhaps detailed mapping of nNOS distribution would help answer such computational questions.

## Summary and conclusion

The basic physiological and pharmacological properties of GABA_A_R transmission onto NHP GCs are virtually identical to those in SDR GCs. In contrast, while EtOH enhances both sIPSC frequency and tonic GABA_A_R currents in nearly all SDR GCs, such enhancement is far less prevalent in NHP GCs, and the prevalence varies considerably across individual NHPs, possibly providing a cellular substrate of variation in behavioral sensitivity to acute EtOH intoxication. Given that low sensitivity to EtOH-induced disruption of cerebellar processing is a risk factor for alcoholism in humans, our results further highlight variation in GC GABA_A_R sensitivity to EtOH as a potential cellular contribution to such risk (Kaplan et al., 2013). Future studies should examine how modulating GC GABA_A_R sensitivity to EtOH influences EtOH intoxication and consumption in both rodent and NHP animal models.

## Methods

Non-human primate (NHP) care and all NHP procedures were approved by the Oregon National Primate Research Center Animal Care and Use Committee at Oregon Health Science University and carried out according to the NIH Guidelines for the Care and Use of Mammals in Neuroscience and Behavioral Research. Euthanasia was carried out according to the American Veterinary Medical Association (AVMA) Guidelines on Euthanasia as detailed in (Daunais et al., 2010) (and below in supporting information). All primates used were euthanized as control subjects in various ongoing studies unrelated to the current study, and no additional primates were euthanized for the purposes of this study.

### Cerebellar slice preparation

For NHPs (6–18 year old males and females), after ketamine sedation, animals were intubated and maintained on a mixture of 1 L O_2_ and room air in order to ensure O_2_ saturation. Sodium pentobarbital was given to establish deep anesthesia during which the calvaria was removed and the brain with dura intact was exposed as previously described (Davenport et al., 2013). The monkeys were then perfused through the heart with cold, oxygenated artificial cerebrospinal fluid and the brain was removed within 5 min after death and prepared for electrophysiology. Supporting Information contains the full details of this procedure. Upon removal of the brain, the cerebellum was rapidly isolated and immersed in ice cold (0–2°C), low-sodium, ACSF composed of sucrose 220, KCl 2, NaH_2_PO_4_ 1.5, MgSO_4_ 1.2, d-glucose 10, NaHCO_3_ 26, and CaCl_2_ 0.2 and transported to the electrophysiology laboratory. The cerebellum was cut into quarter segments, two of which were mounted, parallel to the sagittal plane, in a slicing chamber filled with ice cold (0–2°C), low sodium ACSF. In initial experiments with SDRs (20–60 day old males and females), the methods and solutions for tissue isolation and slicing were exactly as described previously (Rossi and Hamann, 1998; Hamann et al., 2002a; Rossi et al., 2003; Kaplan et al., 2013). However, upon discovering the lack of response to EtOH in NHP GCs, we considered that such differences could result from differences in how we harvested NHP and SDR tissue. Accordingly, we conducted additional experiments in which fully adult SDRs were used (4–6 months old), and animal treatment and tissue harvest were conducted as similarly as possible to NHP tissue harvest. Animals were sedated with Isoflurane and then injected with Ketamine/xylazine mixture (80/12 mg/ml at 8.1 μl/g body weight = 0.6/0.097 mg/g) to deeply anesthetize the animal. The sufficiency of anesthesia was tested by checking paw reflexes and pinching the tail. Surgical access to the heart was implemented, and the animals were transcardially perfused with oxygenated artificial cerebrospinal fluid containing (ACSF) composed of (in mM): NaCl 124, KCl 5, NaH_2_PO_4_ 3, MgSO_4_ 2, d-glucose 10, NaHCO_3_ 26 and CaCl_2_ 2 (290–300 mOsm, 7.3–7.4 pH adjusted by 95% O_2_/5% CO_2_). Removal and extraction of the brain/cerebellum was intentionally delayed (~15 min) to match the timing in primates, and was performed in ice-cold, O2 bubbled, low sodium/sucrose ACSF. In these animals, dose response curves for EtOH were generated and compared to values obtained for NHP GCs and for the original group of SDRs. Similar to the original group of SDRs, all SDR GCs responded to all doses of EtOH tested (25, 52, 79, and 105 mM), and the magnitude of the response to each dose was significantly greater (at least *P* < 0.05 for all doses tested) than the response in NHP GCs, and not significantly different from the response in the original group of SDRs. Accordingly, both sets of SDR values were combined for final analysis/comparison to NHPs and for figure presentation (Figure 3). For all preparations, parasagittal slices (350 μm) were made with a vibrating tissue slicer (Vibratome). Slices were incubated in warmed (33 ± 1°C), normal ACSF [in mM: 124 NaCl, 26 NaHCO_3_, 1 NaH_2_PO_4_, 2.5 KCl, 2.5 CaCl_2_, 2 MgCl_2_, 10 D-glucose, and bubbled with 95%O_2_/5% CO_2_ (pH 7.4).] for 1 h after dissection, then maintained at room temperature until used. Kynurenic acid (1 mM) was included in the holding solution (to block glutamate receptors to reduce potential excitotoxic damage) but was omitted from the experimental solutions.

#### Electrophysiology

Recordings were made from 78 NHP and 71 SDR GCs from 25 and 27 animals, respectively. Slices were placed in a submersion chamber on an upright microscope, and viewed with an Olympus 60× (0.9 numerical aperture) water immersion objective with differential interference contrast and infrared optics. Slices were perfused with ACSF at a rate of ~7 ml/min at room temperature (22–24°C). Drugs were dissolved in ACSF and applied by bath perfusion. Visually identified GCs were voltage-clamped (Vh = −60 mV) with patch pipettes, constructed from thick-walled borosilicate glass capillaries and filled with an internal solution containing (in mM): CsCl 130, NaCl 4, CaCl_2_ 0.5, HEPES 10, EGTA 5, MgATP 4, Na_2_GTP 0.5, QX-314 5. Solutions were pH adjusted to 7.2–7.3 with CsOH. Electrode resistance was 4–10 MΩ. Cells were rejected if access resistance was greater than 15 MΩ. This access resistance rejection threshold, combined with GC input resistances of greater than 2 GΩ means that series resistance errors were always less than 1%, and thus were not compensated. Note, the intracellular [Cl^−^] sets E*_Cl_*- to ~0 mV, which for the holding potentials used in all experiments (−60 mV), results in GABA_A_R currents being inward (downward deflections in displays of the holding current). Accordingly enhancement and block/suppression of tonic GABA_A_R-mediated currents are inward and outward, respectively. eIPSCs were elicited by placing a large patch-electrode (~2 MΩ), filled with ACSF into the slice within a 500 μm of the recorded cell, and increasing the stimulus intensity (of 100 μs duration) until a synaptic response was evoked. Complete blockade by the GABA_A_R antagonist GABAzine (10 μM) was used as confirmation that the response was a GABA_A_R-mediated IPSC. Cells with residual synaptic currents (presumably glutamatergic) we excluded from analysis.

#### Immunocytochemistry

Slices were fixed in 4% paraformaldehyde in phosphate buffered saline (PBS) for 17 h. Slices were then washed and incubated for 40 min in blocking solution [PBS, 0.5% Triton X-100, and bovine serum albumin (0.5 mg/ml)]. Next, they were incubated for 1–2 days with primary antibody in PBS and Triton. Slices were washed three times (10 min each) in PBS, then incubated for 45 min with an Alexa-conjugated secondary antibody. Slices were mounted in Citifluor and imaged with confocal microscopy. See reagents below for source and dilution of antibodies used.

#### Confocal microscopy

Images were acquired with a Zeiss confocal LSM780 laser scanning Microscope, using accompanying Zeiss software for acquisition, processing and subsequent analysis. A laser line falling within 20 nm of the peak absorbance was used for each of the various fluorophores, with appropriate excitation, dichroic and emission filters. Images were acquitted through a 40×, 1.4 N.A. PlanApo oil-immersion objective. Pinhole diameter and slice step thickness were optimized for the objective. For GABA_A_R subunit distribution studies, stacks of image planes were acquired, and then projected into a single stacked image (10 μm thick). Images in Figures 1D,E are representative of others obtained from three separate animals, three slices from each animal, and five distinct regions of each slice.

#### Analysis of GABA_A_R currents

Membrane currents were acquired at 20 kHz, filtered at 10 kHz, and analyzed with pClamp (v.6.3) software (Axon Instruments, Foster City, CA). For analysis and display of sIPSCs, data were filtered at 2–5 kHz. When quantifying sIPSC occurrence, sIPSCs were defined as current deflections that have an amplitude (measured from the mean current) greater than the peak-to-peak amplitude of the current noise and with a decay time constant at least 3-fold slower than the rise time. The tonic current was assessed by fitting the Gaussian distribution of all data points not skewed by synaptic events from a point 3 pA to the left of the peak value to the rightmost (largest) value of the histogram distribution. Drug-induced changes in tonic GABA_A_R current magnitude and sIPSC frequency were calculated by comparing the amplitude/frequency in the drug vs. the mean amplitude/frequency of the currents before and after drug application.

#### Statistics

All data are expressed as the mean ± the standard error of the mean. For statistical comparisons between SDRs and NHPs, unpaired Student's *t*-tests were used, and paired Student's *t*-tests were used for within cell comparisons of effects of drugs on synaptic responses. In all cases, we set the threshold for significance at *P* < 0.05.

#### Reagents

All reagents were from Sigma Chemicals (St. Louis, MO) except gabazine and kynurenic acid (all from Ascent scientific, UK). Primary antibodies were (host/supplier/dilution): α6 subunit of GABA_A_R (rabbit/Millipore AB5610/1:200), δ subunit of GABA_A_R (rabbit/Millipore AB9752/1:200), nNOS (rabbit/Cayman chemicals 160870/1:200), and Calbindin D-28K (Rabbit/Chemicon/1:2000). Secondary antibodies were various excitation maxima Alexafluors (Invitrogen) from appropriate hosts diluted 1:500. For some antibodies (α6 and δ subunit of GABA_A_R), specificity has been confirmed by lack of labeling in relevant knockout mice, for others (Calbindin) specificity has been confirmed by their labeling of a single band on western blots (see manufacturer website for details, and links therein). For those antibodies where such confirmation of specificity was not already available (nNOS), we confirmed specificity ourselves, by examining slices from nNOS knockout mice (*Nos1^tm1Plh^* homozygotes, backcrossed to C57BL/6J mice for > 10 generations, from Jackson Laboratory), which did not display detectable nNOS staining (Kaplan et al., 2013). Furthermore, for all of the antibodies we used, the general qualitative expression pattern within the cerebellum is similar to what has been shown with other antibodies in other reports and to our own studies with alternative antibodies. Importantly, for all of the crucial immunohistochemical studies (GABA_A_R subunits), we have conducted parallel electrophysiological studies of the relevant proteins' activity which confirm our histochemical assessments (see electrophysiological experiments in Figure 1 for functional confirmation of GABA_A_R subunit expression levels).

## Supporting information

### Monkey necropsy

Monkey protocols were approved by the Oregon National Primate Research Center Animal Care and Use Committee and were carried out in Hillsboro, OR. Monkeys were sedated with ketamine (15 mg/kg, i.m.) and hair was removed from the head, neck and calves. A catheter was inserted into the saphenous vein and secured to the skin with tape. After flushing with saline, a surgical plane of anesthesia was established by slowly injecting to effect 20–35 mg/kg sodium pentobarbital through the intravenous catheter and flushing with an equal volume of saline. The adequacy of surgical anesthesia was verified by the absence of the corneal, palpebral, and hindlimb withdrawal reflexes. Cardiac and respiratory function and blood pressure were closely monitored throughout the procedure by veterinarians. Incisions were made in the scalp along the sagittal suture from bregma to the 3rd cervical vertebra and perpendicularly along the coronal suture and occipital ridge, after which the skull was exposed by reflecting the temporal, frontal, and occipital muscles, by reflecting the muscles along the sagittal suture and the occipital ridge. The craniotomy was carried out using bone rongeurs after drilling a 1-cm diameter hole in the right parietal bone. Once the bone was removed, the calvaria was removed from the dura mater and the occipital ridge was removed. The exposed brain was gently covered with saline-soaked gauze. The animal was turned onto its back and sequential incisions were made through the skin from the clavicle to the pelvis and then through the abdominal and pectoral muscles and diaphragm. The ribcage was cut and the pericardium was opened. The right atrium was cut to drop blood pressure and a 16-gauge cannula was inserted into the ascending aorta and clamped into place. The descending aorta was clamped in the lower thorax. The animal was perfused with 1.5 L of oxygenated artificial cerebrospinal fluid containing (ACSF) in 2 min composed of (in mM): NaCl 124, KCl 5, NaH_2_PO_4_ 3, MgSO_4_ 2, d-glucose 10, NaHCO_3_ 26 and CaCl_2_ 2 (290–300 mOsm, 7.3–7.4 pH adjusted by 95% O_2_/5% CO_2_). Following perfusion, the animal was immediately turned over and the dura mater was cut at the posterior extent of the occipital lobes and along the midsagittal line and reflected. The falx cerebri and tentorium cerebelli were removed and the occipital lobes were elevated with a scalpel handle. The cervical cord was cut at C3 using a scalpel and the cranial nerves were bluntly dissected from the brain using the scalpel's handle. The brain was removed from the skull in a caudal to rostral fashion. The brainstem was separated using a razor blade by sectioning just rostral to the ponto-medullary junction and by sectioning the cerebellar peduncles. The tissue block was submerged in a small beaker of oxygenated, low-sodium, ice-cold ACSF composed of sucrose 220, KCl 2, NaH_2_PO_4_ 1.5, MgSO_4_ 1.2, d-glucose 10, NaHCO_3_ 26, and CaCl_2_ 0.2 and transported to the electrophysiology laboratory. Once the cerebellum was isolated, the remaining brain and tissues of the body were prepared for additional studies by other investigators.

## Author contributions

Claudia Mohr, John Welsh, Kathleen A. Grant, James B. Daunais, Joshua S. Kaplan, and David J. Rossi designed the experiments. James B. Daunais performed the necropsies. Claudia Mohr, Joshua S. Kaplan, and David J. Rossi performed the electrophysiology experiments and Olena Kolotushkina performed the immunocytochemistry experiments. Claudia Mohr, John Welsh, Kathleen A. Grant, Joshua S. Kaplan, and David J. Rossi analyzed the data and wrote the manuscript.

### Conflict of interest statement

The authors declare that the research was conducted in the absence of any commercial or financial relationships that could be construed as a potential conflict of interest.
